# Red Light/Green Light, a Dual Fluorescent Protein Reporter System To Study Enhancer-Promoter Specificity in *Drosophila*

**DOI:** 10.1534/g3.119.401033

**Published:** 2020-01-03

**Authors:** Eric M. Camino, Micheal L. Weinstein, Mary P. List, Jordan E. Vellky, Mark Rebeiz, Thomas M. Williams

**Affiliations:** * Department of Biology and; ‡ The Integrative Science and Engineering Center, University of Dayton, Dayton, OH 45469, and; † Department of Biological Sciences, University of Pittsburgh, Pittsburgh, PA 15260

**Keywords:** enhancer, remote control element, promoter, tethering element, Drosophila

## Abstract

Enhancers activate gene transcription in spatial and temporal patterns by interactions with gene promoters. These elements typically reside distal to their target promoter, with which they must interact selectively. Additional elements may contribute to enhancer-promoter specificity, including remote control element sequences within enhancers, tethering elements near promoters, and insulator/boundary elements that disrupt off-target interactions. However, few of these elements have been mapped, and as a result, the mechanisms by which these elements interact remain poorly understood. One impediment is their method of study, namely reporter transgenes in which enhancers are placed adjacent to a heterologous promoter, which may circumvent mechanisms controlling enhancer-promoter specificity and long-range interactions. Here, we report an optimized dual reporter transgene system in *Drosophila melanogaster* that allows the simultaneous comparison of an enhancer’s ability to activate proximal and distal fluorescent reporter genes. Testing a panel of fluorescent transgenes *in vivo*, we found a two-protein combination that allows simultaneous measurement with minimal detection interference. We note differences among four tested enhancers in their ability to regulate a distally placed reporter transgene. These results suggest that enhancers differ in their requirements for promoter interaction and raise important practical considerations when studying enhancer function.

The precise spatial and temporal patterning of gene expression is a fundamental feature of embryonic development (Davidson and Erwin 2006; Lagha *et al.* 2012; Peter and Davidson 2015). These patterns of expression are governed by enhancer elements within *cis*-regulatory regions that direct the initiation of transcription at the promoter of a regulated gene (Levine and Davidson 2005; Lagha *et al.* 2012). The *cis*-regulatory regions of metazoan genes are notoriously vast and complicated (Wray *et al.* 2003; Nelson *et al.* 2004), where some enhancers are located near the promoter region of a gene (proximal) or are positioned far away (distal) (Kvon *et al.* 2014). For example, the enhancer that drives Sonic hedgehog (*Shh*) expression in the organizing center of the mouse embryonic limb bud resides over 1 Megabase (Mb) away from the *Shh* promoter within the *lmbr* gene locus (Lettice *et al.* 2003). Therefore, this enhancer must identify and interact with the *Shh* promoter through the formation of a chromosomal loop (Amano *et al.* 2009). Similarly, active expression of β-globin cluster genes in erythroid cells requires long-range interactions between the β-globin Locus Control Region (LCR) and the promoters of expressed genes by interactions between proteins bound to these two type of regulatory elements (Tolhuis *et al.* 2002; Deng *et al.* 2012). Although *Shh* and *β-globin* genes have provided detailed examples of long-distance regulation, it remains challenging to find the DNA sequences involved in other cases of enhancer-promoter interactions.

High-throughput studies characterizing looping conformations between gene promoters and distal regulatory sequences are indicative that long distance gene regulation is common (Sanyal *et al.* 2012; Dekker and Misteli 2015; Dekker and Mirny 2016). This form of regulation is not only relevant to development, but the consequences of mutations in these interacting sequences can have effects on health. For example, the human *FTO* locus harbors a nucleotide variant that prevents an enhancer from activating the genes *Irx3* and *Irx5* that are located at a distance of ∼0.5 Mb and 1 Mb respectively. The loss of expression of these genes results in increased white adipocytes, which is associated with obesity (Claussnitzer *et al.* 2015). Long distance regulation has evolutionary implications, as several evolved patterns of gene expression were traced to enhancers located distally from their target promoters (Martin and Orgogozo 2013). This includes enhancers that control derived gene expression patterns for diverse fruit fly pigmentation traits (Jeong *et al.* 2008; Williams *et al.* 2008; Camino *et al.* 2015; Koshikawa *et al.* 2015), an evolved pattern of human neocortical gene expression (Boyd *et al.* 2015), the persistence of lactase expression in humans (Tishkoff *et al.* 2007), and a pattern of expression that shaped the domestication of maize (Studer *et al.* 2011) among others.

With the broad importance of long-distance gene regulation to development, health, and evolution, it is important to understand the mechanisms involved in establishing interactions between enhancers and promoter regions. Seminal studies identified several types of sequences that facilitate these interactions. One is a tethering element (Teth), which can reside proximally to a transcription start site and that is required for interaction with a distal enhancer (Figure 1A) (Calhoun *et al.* 2002; Calhoun and Levine 2003). A second type of sequence is referred to as a remote control element (RCE), a type of sequence embedded within an enhancer and which is necessary for the enhancer to interact with a distal promoter (Figure 1B) (Swanson *et al.* 2010). Specific elements within the core promoter have also been shown to have significant roles in determining enhancer-promoter specificity (Butler and Kadonaga 2001).

**Figure 1:**
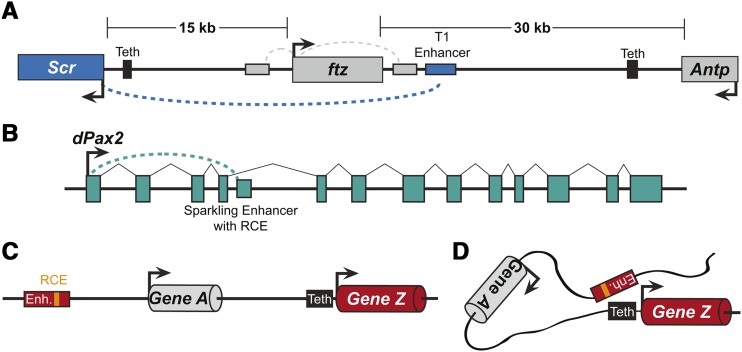
Gene regulation via long distance enhancer-promoter interactions. (A) A short repeat motif sequence known as a “Tethering Element” (Teth) located in a promoter-proximal region can facilitate interaction with a distal enhancer. The T1 Enhancer bypasses the proximal *ftz* gene promoter to interact with the *Scr* gene promoter that is >15 kilobase pairs (kb) away (Calhoun *et al.* 2002). (B) A “remote control element” (RCE) sequence within the Sparkling Enhancer is required to activate the cone cell pattern of expression seen for the *dPax2* gene. Sparkling resides in the 4^th^ intron of *D. melanogaster dPax2* gene (Swanson *et al.* 2010). (C-D) With many enhancers (Enh.) located at a distance from their target promoters (C) it is possible that remote control elements and tethering elements represent a common feature of gene regulation to bring distantly-located enhancers into close proximity to a target promoter (black arrow) to activate gene expression (D).

The broad relevance of tethering elements and enhancer-embedded remote control elements remains unknown, as these sequences are seldom sought out, and not identifiable by conventional methods. Typically, reporter transgene assays test an enhancer sequence placed immediately 5′ of a minimal heterologous promoter for which no choice of promoter is provided (Barolo *et al.* 2004; Swanson *et al.* 2010). This architecture eliminates any requirement for a looping interaction. Furthermore, this canonical configuration would not detect the effects of mutations in remote control elements, masking an entire class of potential regulatory variation. Thus, we sought to develop a reporter transgene vector system that may simultaneously assess the capability of enhancers to regulate both proximal and distal reporter genes. This allows one to assay the ability of an enhancer to communicate with a promoter over a distance and presents a platform in which sequences necessary for such communication can be identified. Here we report the optimization of such a system for use in transgenic *Drosophila* (*D*.) *melanogaster* and test this system with four different enhancers, two heterologous promoters, and one endogenous promoter region.

## Materials and Methods

### Generating pRLGL vectors

The vector backbone used for construction of the dual reporter system was the mS3aG reporter vector (Camino *et al.* 2015), a derivative of the S3aG vector that lacks *Bgl*II sites. We synthesized two cassettes, a 1,296 base pair (bp) cassette flanked by *Eco*RI and *Asc*I restriction enzyme sites, and a 2,014 bp cassette flanked by *Asc*I and *Age*I sites. The *Eco*RI*-Asc*I cassette possessed the DsRed.T4-NLS reporter transgene (Barolo *et al.* 2004) that had its *Sbf*I site removed by a synonymous mutation, and which is flanked by a 5′ *hsp70* core promoter (flanked by *Asc*I and *Stu*I sites) and 3′ polyadenylation signal (flanked by *Eco*RI and *Fse*I sites). The *Asc*I*-Age*I cassette contained the core region of the enhancer known as the dimorphic element (Williams *et al.* 2008; Rogers and Williams 2011; Rogers *et al.* 2013) flanked by *Asc*I and *Sac*II sites on one side, and *Nhe*I and *Sbf*I sites on the other, a 1 kilobase pair (kb) spacer sequence taken from the *bab1* 1^st^ intron for which every other bp was replaced with a non-complementary transversion mutation, and an *Hsp70* promoter with 5′ *Bgl*II and *Bam*HI sites and 3′ *Xho*I and *Age*I sites. The mS3aG vector was opened at its *Eco*RI and *Age*I sites and the two synthesized cassettes were inserted to complete our first-generation red light/green light vector where the *EGFP-NLS* reporter gene was displaced from the dimorphic element enhancer by 1 kb.

**Figure 2:**
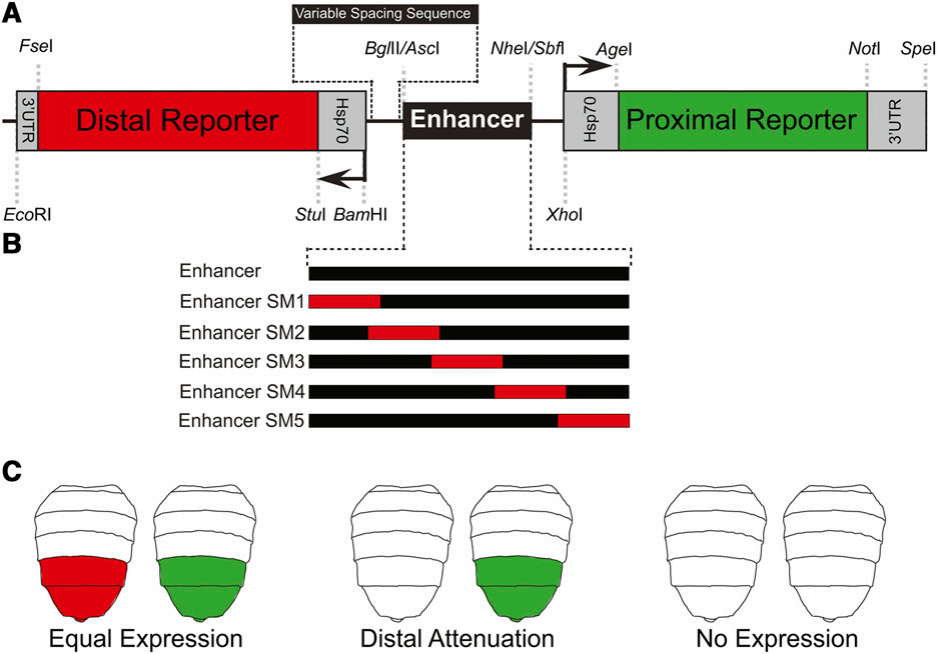
Design of the Red Light Green Light dual reporter transgene system. (A) An enhancer can be situated between green and red fluorescent reporter genes each with a minimal promoter. The red fluorescent reporter can be positioned distal to the enhancer by the inclusion of spacer sequences of 1, 2, 4, and 8 kilobase pairs (kb). The enhancer, promoter, reporter, and 3′UTR sequences are all flanked by unique restriction endonuclease sites allowing any of these sequences to be readily replaced with another sequence; gray dashed lines represent the locations of unique restriction enzyme sites. (B) Representation of scanning mutagenesis approach that can be used to identify remote control element-like sequences in an enhancer. The red blocks represent sequences that have been modified by introduced mutations. (C) An example of potential outcomes for reporter gene expression in the dual-reporter system for an enhancer that is active in the *D. melanogaster* posterior abdomen. Type 1 – Equivalent fluorescent protein expression from both the proximal and distal reporters, indicating that the enhancer can regulate the reporter gene at a distance. Type 2 – Expression is seen solely for the proximal reporter gene, this indicating that the enhancer or its mutant form is not capable of activating the distal reporter gene. Type 3 – No expression is observed for either reporter gene, which would be anticipated when a mutation destroyed a sequence necessary for the enhancer’s spatial or temporal domains of regulatory activity.

To vary the spacing between the *DsRed.T4-NLS* reporter gene and the dimorphic element, we synthesized a *Stu*I*-Age*I cassette that possesses an *Hsp70* promoter flanked by *Stu*I and *Bam*HI sites, a 2 kb spacer sequence flanked by *Bam*HI and *Bgl*II sites, the dimorphic element core enhancer flanked by *Asc*I and *Sac*II sites on one side and *Nhe*I and *Sbf*I sites on the other, and a second *Hsp70* promoter flanked by *Xho*I and *Age*I sites. The 2 kb spacer sequence was derived from a tandem duplicate 1 kb sequence from the *bab1* 1^st^ intron, where the first and second kb of sequence were respectively altered at odd and even bp by non-complementary transversions, and for which *Nhe*I, *Sbf*I, *Spe*I, *Sac*II, and *Age*I sites were eliminated by bp substitutions. The *Stu*I*-Age*I cassette from the first-generation vector was removed and replaced with this 3,103 bp *Stu*I*-Age*I cassette. The resulting vector with the 2 kb spacer and the dimorphic element was named pRLGL2+DEcore (Figure 2A).

The 0 kb spacer version (pRLGL0+DEcore) was created by removing the 2 kb spacer from the pRLGL2+DEcore vector by BamHI and BglII digestion and subsequent re-ligation. A 1 kb spacer sequence was PCR amplified from the 2 kb spacer template using the RLGL2 1K spcF1 (TTTCCGggatccGCGCAACAACGCAGCTGGGTAGCG) and RLGL2 1K spcR1 (TTGCCagatctGTGTATCCGTCCCAGTACCTCG) primers which added flanking *Bam*HI and *Bgl*II sites. This PCR product was cloned into the *Bam*HI and *Bgl*II sites of the pRLGL2+DEcore vector replacing the 2 kb spacer, and making the vector pRLGL1+DEcore. The 4 kb spacer vector, pRLGL4+DEcore, was generated by In-Fusion Cloning (Clontech Laboratories Inc.) of a second 2 kb spacer with flanking *Bgl*II sites into the *Bgl*II site of pRLGL2+DEcore. The second 2 kb spacer sequence was derived from a synthesized tandem duplicate 1 kb sequence from the *bab1* 1^st^ exon, where the first and second kb of sequence were respectively altered at odd and even positions by non-complementary transversions, and for which all TTTAT (Abd-B binding motifs) were altered to TTGGG. The synthesized spacer piece was subsequently PCR-amplified using the primers In-Fusion (15) Spacer Fwd (CTGCCCGCCCAGATCTGGATTGTCAGCGTGTACACC) and In-Fusion (15) Spacer Rvs (ATGGCGCGCCAGATCTTCTCGGTACCCAATCTAATAAGC). The 8 kb spacer vector, pRLGL8+DEcore, was generated by In-Fusion cloning a synthesized 4 kb spacer sequence into the pRLGL4+DEcore vector at the *Bam*HI site located at the 5′ end of the *Hsp70* promoter for the *DsRed.T4-NLS* reporter gene. The spacer sequences were derived from a 4,000 bp sequence from the *bab1* 1^st^ intron that had its nucleotide composition scrambled, substituted with non-complementary transversion at odd base pairs, had Hox-like binding motifs (YTAATKV and TTTAT) mutated, and that had restriction enzyme sites removed that are utilized as single cutters elsewhere in the pRLGL8+DEcore vector. The spacer sequence was synthesized (GenScript Inc.) and subsequently PCR-amplified for In-Fusion cloning using the primers InFus (15) 8k Fwd (CCGGCGCTCGGATCCGCTTTCTTAAGTAGTACGC) and InFus (15) 8k Rvs (CTCTGTAACTGGATCTTCAGTTCAAAACAATCGTTC).

### The design of alternate fluorescent reporter transgene plasmids

The sequence between the *Age*I and *Spe*I sites of the S3aG (Rogers and Williams 2011) vector containing the yBE0.6 enhancer (Camino *et al.* 2015) was used as a starting point for the substitution of various fluorescent protein-coding sequences in place of that for *EGFP-NLS* with the in-frame nuclear localization signal (NLS) sequence of the *tra* gene (Hedley *et al.* 1995), and a polyadenylation (polyA) signal in the 3′ untranslated region (UTR). This reporter gene and 3′UTR cassette was removed by AgeI and SpeI (NotI in the case of *mCerulean-NLS*) digestion and replaced by cassettes containing the coding sequences for other fluorescent proteins. This included the protein-coding sequence for mCherry, which was based upon the sequence in the pmR-mCherry vector (Clontech Inc.). The FASTA format sequence from the 1^st^ codon of *mCherry* to the last codon amino acid was combined 5′ and in-frame of the coding sequence for the *tra* gene nuclear localization signal and a poly adenylation signal-containing 3′UTR. This coding sequence possessed single instances of *Stu*I and *Sbf*I sites, two restriction enzymes present in multi-cloning sequences of S3aG and pRLGL-type vectors. These sites were therefore removed by substituting a single synonymous base change. The *E2-Crimson* fluorescent protein (Strack *et al.* 2009) coding sequence was obtained from the sequence file for the pCMV-E2-Crimson vector (Clontech Inc.) and the FASTA format sequence was grafted 5′ and in-frame of the *tra* NLS sequence. The *Sbf*I site that resided within the *E2-Crimson* sequence was destroyed by substituting a synonymous mutation. The *mCerulean* protein coding sequence was obtained from the CMV-Brainbow-1.0L vector (Addgene plasmid #18721), and the text was inserted in front of and in-frame with the text for *tra* NLS by *Age*I and *Not*I restriction sites. These sequences were synthesized (GenScript Inc.) and cloned into the S3aG+yBE0.6 vector backbone after removal of the *EGFP-NLS* cassette, creating S3 am Cherry-NLS+yBE0.6, S3 am Cerulean-NLS+yBE0.6, S3aE2-Crimson-NLS+yBE0.6.

### Generating pFRGL vectors

A reporter gene sequence was designed for the pFRGL (Far Red/Green Light) vector composed of an *Hsp70* promoter, *E2-Crimson-NLS* coding sequence, and a poly adenylation signal-containing 3′ UTR. This transgene was flanked by a 5′ *Bam*HI site and 3′ *Eco*RI site. This sequence was synthesized and cloned in place of the distal reporter from the 2, 4, and 8 kb spacer vectors with the dimorphic element core enhancer. The 2 kb spacer was removed by BamHI and BglII digestion followed by re-ligation to create the version lacking a spacer. These E2-Crimson-NLS containing vectors are referred to as pFRGL0+DEcore, pFRGL2+DEcore, pFRGL4+DEcore, and pFRGL8+DEcore.

### Cloning of enhancer elements and promoter elements

The enhancer DNAs were obtained from *D. melanogaster* genomic DNA (strain 14021‐0231.04) that was acquired from the San Diego Drosophila species stock center. Enhancers were PCR-amplified from the genomic DNA using the primer combination shown in Table 1, which added *Asc*I and *Sbf*I sites. These PCR amplified enhancers were cloned into the *Asc*I and *Sbf*I sites of the pRLGL8+DEcore vector in place of the dimorphic element core enhancer.

**Table 1:** Primers used to clone *D. melanogaster enhancers* for cloning into reporter transgene vectors

CRE	∼Size	Primer	Sequence
yBE0.6	600	BE2.5 Fwd	TTCCGggcgcgccCTGTGGGTGCAATGATTTAGAATG
		BE3.5 Rvs	TTGCCcctgcaggGTTATTGGCAGGTGATTTTGAGC
t_MSE2	350	tan_MSE-mid-F	TTCCGggcgcgccTGAAATAATAATAAATAATCAGAAT
		tan_MSE-mid-R	TTGCCcctgcaggTGTTTCAACTCAATCCTAGCAGTTGG
dimorphic	690	DE core Fwd	TTCCGggcgcgccTCGCCTccgcggCTCTTTCTCTTTGCCATTTTAAC
element core		DE core Rvs	TTGCCcctgcaggCCCTTGgctagcGTGTGTGAACCAATTTGTTGTGC
LAE	1,400	bab TRE Fwd	TTCCGggcgcgccGTGAGGGGCAAATTATGGAGAG
		bab TRE Rvs	TTGCCcctgcaggGTGCGCCTAACTAGCCAACAATTAG
Expanded Dimorphic	1,580	sub1orthoF1	TTCCGggcgcgccCACATAAAAATCAGCAACAAASTTGC
Element		dimorphic Rvs1	TTGCCcctgcaggCAAAACKGCRCATAAAAMSAAATTACA

Notes:

1. ‘ggcgcgcc’ and ‘cctgcagg’ are sequences recognized respectively by the AscI and SbfI restriction endonucleases. These restriction enzyme sites were used to clone PCR amplified sequences into the pRLGL8 reporter vector. ‘ccgcgg’ and ‘gctagc’ are sequences recognized respectively by the SacII and NheI restriction endonucleases.

2. The approximate PCR product sizes are reported in base pairs (bp).

3. ‘S’, ‘K’, ‘R’, and ‘M’ are IUPAC notations for degenerate bases. S = G or C, K = G or T, R = A or G, and M = A or C.

The *Drosophila* synthetic core promoter or DSCP (Pfeiffer *et al.* 2008) was PCR-amplified from genomic DNA from flies of the Bloomington Drosophila Stock Center stock ID#41269 that possess a transgene with this core promoter. The primers utilized were DSCP Infus Fwd (AAGGGCGAATTAAACAGGCCTGTTTGGTATGCGTCTTGTGATTC) and DSCP Infus Rvs (ACTACTTAAGAAAGCGGATCCGAGCTCGCCCGGGGATCGAG) which added *Bam*HI and *Stu*I restriction enzyme sites for cloning the amplified promoter in place of the *Hsp70* promoter 5′ of the *E2-Crimson-NLS* transgene of the pFRGL8+DEcore vector, creating the pFRGL8+DEcore+DSCP vector. A 1,157 bp sequence containing the *bab2* promoter and proximal region was synthesized and subsequently cloned in place of the same *Hsp70* promoter of the pRLGL8+DEcore vector by GenScript Inc., creating the vector pRLGL8+DEcore+bab2p.

### Generating control vectors

To create a version of the pRLGL8 vector without (w/o) an enhancer, we digested the pRLGL8+DEcore vector with the AscI and AgeI restriction enzymes, which removed a cassette containing the dimorphic element enhancer and the *Hsp70* promoter for the *EGFP-NLS* reporter gene. The *Hsp70* promoter was then PCR amplified with primers that added a 5′ *Asc*I (TTCCGggcgcgccCTCGAGGAGCGCCGGAGTATAAATAGAGG) site and a 3′ *Age*I (TTTGCCaccggtGGATCGTTTAAACAGGGCTCTCGAC) site. Following PCR and enzyme digestion, this promoter was ligated into the *Asc*I and *Age*I sites of the digested vector, creating the vector referred to as pRLGL8 w/o enhancer.

To create a version of the pRLGL8 vector w/o the proximal *Hsp70* promoter for the *EGFP-NLS* reporter gene, we used the AscI and AgeI digested pRLGL8+DEcore vector that lacked the dimorphic element enhancer and the *Hsp70* promoter for the *EGFP-NLS* reporter gene. The dimorphic element was then PCR amplified with primers that added a 5′ *Asc*I (TTCCGggcgcgccTCGCCTccgcggCTCTTTCTCTTTGCCATTTTAAC) site and a 3′ *Age*I (TTTGCCaccggtGTGTGTGAACCAATTTGTTGTGC) site. Following PCR and enzyme digestion, this enhancer was ligated into the AscI and *Age*I sites of the digested vector, creating the vector referred to as pRLGL8+DEcore w/o proximal promoter.

### Transgenic creation and D. melanogaster integration

The pRLGL, pFRGL, and S3aG-based reporter vectors with various fluorescent protein coding sequences were site-specifically integrated into the *D. melanogaster* germline attP40 landing site (Markstein *et al.* 2008) on the 2^nd^ chromosome (Best Gene Inc.) by a phiC31 integrase approach (Groth *et al.* 2004). Transgenic *D. melanogaster* were maintained at 22° and with a Sugar Food medium recipe (Salomone *et al.* 2013).

### Analysis of fluorescent reporter output by confocal microscopy

The yBE0.6 enhancer was used to drive expression of the newly designed fluorescent reporters. This enhancer’s activity was assessed at ∼85 hr after puparium formation (hAPF) (Camino *et al.* 2015). The developmental time point used for the expression analysis of pRLGL and pFRGL transgenes were ∼70 hAPF for those with the dimorphic element core enhancer, expanded dimorphic element, and leg and antennal enhancer (Baanannou *et al.* 2013; Rogers *et al.* 2013); ∼85 hAPF for the yBE0.6 enhancer containing vectors, and ∼95 hAPF for the t_MSE2 enhancer containing vectors (Camino *et al.* 2015). An Olympus Fluoview 1000 confocal microscope was used to capture projection images from whole mount pupae. The microscope settings were optimized and are presented in Table 2 and Table 3.

**Table 2:** Confocal microscope settings for imaging transgenic *D. melanogaster* with S3a-series+yBE0.6 enhancer fluorescent protein reporter transgenes

Fluorescent Protein	EGFP	mCerulean-NLS	mCherry-NLS	E2-Crimson-NLS
**Laser (nm)**	488	458	543	633
**Laser%**	10	20	20	15
**HV**	700	850	850	750
**Gain**	1	2	1	1
**Offset**	1	20	10	5
**Aperture**	200	200	200	200
**Step Size (μm)**	10	10	10	10
**Excitation Filters**	485-595 (512)	450-585 (476)	550-648 (580)	620-780 (665)
**Emission Filters**	500-530	462-485	555-625	Far Red

**Table 3:** Confocal microscope settings for imaging transgenic *D. melanogaster* with Red Light/Green Light-series dual reporter transgenes

Fluorescent Protein	EGFP	DsRed.T4-NLS	E2-Crimson-NLS
**Laser (nm)**	488	543	633
**Laser%**	10	15	15
**HV**	600	750	700
**Gain**	1	1	1
**Offset**	1	1	5
**Aperture**	200	200	200
**Step Size (μm)**	10	10	10
**Excitation Filters**	485-595 (512)	550-648 (580)	620-780 (665)
**Emission Filters**	495-530	575-640	Far Red

### Figure Construction

*AntP*, *Scr*, *ftz*, *dPax2*, *tan*, *yellow*, and *bab* gene loci representations were obtained from the GenePalette software tool (Rebeiz and Posakony 2004; Smith *et al.* 2017). Confocal projection images were edited using Adobe Photoshop CS3. Figures were assembled with Adobe Illustrator CS3. All confocal projection images were edited by the same set of modifications. Images were rotated to be oriented vertically, specimens were cropped to have the same size dimensions, background levels were set to black, artifacts from area surrounding images were erased with the eraser tool, and images were flattened.

### Data availability

All of the relevant DNA sequences and the encoded fluorescent proteins can be respectively found in Supplementary Documents 1 and 2. All custom DNA sequences were synthesized by GenScript Inc. Plasmid vectors will be made available from the Addgene non-profit vector repository (https://www.addgene.org/). Transgenic fruit fly lines are available upon request. Supplemental material available at figshare: https://doi.org/10.25387/g3.10299482.

## Results

### A dual reporter transgene system to study promoter choice and long-distance gene regulation

To facilitate the identification of tethering elements and remote control elements we constructed a dual fluorescent reporter system called Red Light/Green Light that can simultaneously test the regulatory capability of enhancers when they are situated proximal to one fluorescent reporter gene and distal to a second (Figure 2A). Our initial design of this system used EGFP-NLS and DsRed.T4-NLS However, in further iterations (see subsequent sections below), the DsRed reporter is replaced with the far-red shifted E2-Crimson-NLS which does not interfere with EGFP-NLS detection.

In these Red Light/Green Light vectors, an enhancer can be cloned into the *Asc*I and *Sbf*I restriction enzyme sites located between the coding sequences for two fluorescent reporter genes (Figure 2A). Both the proximal and distal reporter genes possess a *D. melanogaster Hsp70* minimal promoter, but specific restriction sites facilitate the cloning of custom or endogenous promoters. The coding sequence of the proximal reporter is the *enhanced green fluorescent protein* (*EGFP*) gene in-frame with the coding sequence for the nuclear localization signal (NLS) of the *tra* gene on the 3′ end (Hedley *et al.* 1995), which we refer to as *EGFP-NLS*. For the distal reporter gene we initially used the *DsRed.T4-NLS* coding sequence that similarly includes a 3′ sequence for the *tra* NLS (Barolo *et al.* 2004). In this vector (called pRLGL0) the enhancer is located at an equal distance to the promoter of each reporter gene. We created modified versions that possessed an added spacer sequence (with no intended regulatory function) of 1, 2, 4, and 8 kilobase pairs (kb) between the *DsRed.T4-NLS* gene and the enhancer site. These vectors are called pRLGL1, pRLGL2, pRLGL4, and pRLGL8. The spacer sequences used in these vectors originated as *bab1* intron and exon sequences for which every other bp was changed to their non-complimentary transversion. Additionally, ectopic restriction sites and Hox-like transcription factor binding sites were removed from these sequences by bp substitutions to facilitate cloning and detection of enhancer activities with minimal interference from the spacers (see methods and Supplementary Document 1).

The vectors with the added spacer sequence allow for the simultaneous testing of an enhancer’s regulatory activity on proximally and distally located promoters. Moreover, the distal *Hsp70* promoter is flanked by unique *Stu*I and *Bam*HI restriction sites, so this promoter can either be replaced by another promoter or supplemented with additional promoter proximal sequences. It should also be noted that the distal 3′ UTR can be removed by the flanking *Eco*RI and *Fse*I restriction endonuclease sites in the event that another 3′ UTR or mutated version needs to be tested.

We initially sought to determine whether a particular enhancer can mediate its characteristic regulatory activity upon both proximal and distal reporter genes, resulting in equal reporter expression (Figure 2C). It is also conceivable that an enhancer would only be capable of activating expression of a proximal reporter gene, causing distal-attenuated expression (Figure 2C). If distal attenuation occurs, then modified vectors could be made that include additional sequences to find those that rescue long distance gene expression regulation. This could involve replacing the heterologous *Hsp70* promoter with the endogenous promoter of the gene that the enhancer regulates, adding endogenous promoter proximal sequence next to the distal promoter, or testing an expanded enhancer region.

Once a reporter configuration has been identified, where the regulatory activity is imparted effectively upon the distal reporter, it is then feasible to introduce enhancer mutations to map the sequences required for remote control element activity. For instance, a series of scanning mutant enhancers could be made where each mutant includes a unique block of base pairs that were altered by non-complementary transversions (Figure 2B). These mutant versions could then be evaluated for the capability to regulate the expression of the proximal and distal reporter genes. Mutations altering non-functional sequences should lead to an equal reporter expression outcome. Mutations altering sequences that encode aspects of the enhancer’s spatial and temporal regulatory activities should result in the absence of both proximal and distal reporter expression (Figure 2C). Cases in which only the distal reporter’s expression is attenuated, would indicate that the introduced mutations specifically disrupted a remote control element. A similar mutagenesis approach could be applied to promoters and promoter proximal sequences in order to identify sequences functioning as tethering elements.

### Testing the effects of the spacing between an enhancer and a distal reporter gene

**Figure 3:**
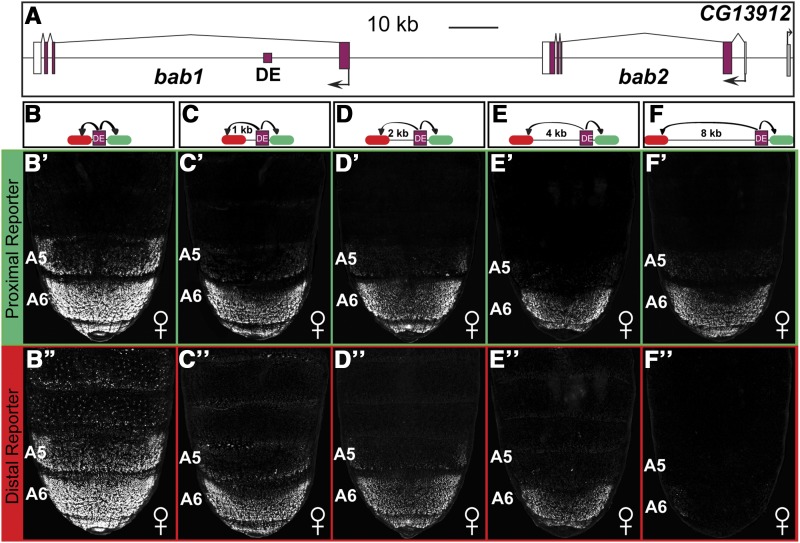
The effects of enhancer-promoter spacing on the expression of proximal and distal reporter genes. (A) *bab* locus showing the relative position of the dimorphic element (DE) core enhancer from the promoters (black arrows) for the *bab1* and *bab2* genes. (B-F) Schematics of the evaluated reporter transgenes. Here, the distance of the dimorphic element from the Ds.Red.T4.NLS (red oval) reporter was altered by the inclusion of spacer sequence, while the EGFP-NLS (green oval) reporter’s position did not change. (B’-F’) Green fluorescence and (B’’-F’’) red fluorescence detected from reporter transgenes with the proximal EGFP-NLS reporter at a consistent distance from the dimorphic element and a DsRed.T4-NLS reporter positioned at varying distances of 0, 1, 2, 4, and 8 kilobase (kb) pairs from the dimorphic element.

In a typical reporter transgene, enhancers are placed immediately adjacent to a heterologous promoter, such as the *Hsp70* promoter of *D. melanogaster* (Rebeiz and Williams 2011). However, few studies have systematically evaluated the effect that distance between an enhancer and promoter exerts on the ability to activate reporter gene expression. We decided to evaluate the regulatory activity of the enhancer known as the dimorphic element of the *bric-à-brac (bab)* gene complex (Williams *et al.* 2008; Rogers *et al.* 2013) (Figure 3A) at various distances between the fluorescent reporter genes (Figure 3B-3F). This enhancer drives the expression of a proximally located fluorescent reporter in the dorsal epidermis of the A5-A7 abdominal segments of transgenic female *D. melanogaster* pupae (Williams *et al.* 2008). In this experiment, we manipulated the distance of this enhancer from the *Hsp70* promoter of the distal DsRed.T4-NLS reporter. When there was no spacer sequence between the enhancer and the distal reporter, we observed robust green and red fluorescence (Figure 3B’ and 3B’’). Moreover, when EGFP-NLS and DsRed.T4-NLS fluorescence were merged widespread co-localization was evident at the single-cell level (Figure S1). This result indicates that the dimorphic element can activate both reporter genes simultaneously. However, when spacers of 1, 2, 4, and 8 kb were included between the dimorphic element and the *DsRed.T4-NLS* reporter gene, we saw a progressive reduction in red fluorescent protein expression (Figure 3C’’-3F’’). Notably, there was little to no expression observed when an 8 kb spacer was used (Figure 3F’’). This suggests that 8 kb of spacer sequence was a sufficient impediment to a functional interaction between the dimorphic element and the distal *Hsp70* promoter. Interestingly, we observed a progressive, albeit less severe, reduction in green fluorescence (Figure 3B’-3F’). This decline in green fluorescence occurred even though the distance between the dimorphic element and the proximal *Hsp70* promoter remained constant. One possible explanation for this outcome is that some of the expressed DsRed.T4-NLS protein emits green fluorescent light rather than red. This possibility is supported by previous findings that some DsRed protein is trapped in a green fluorescent light emitting form (Baird *et al.* 2000). Importantly though, these data show that an 8 kb spacer sequence was suitable to interrupt the communication of the dimorphic element with a heterologous promoter in a *D. melanogaster* transgene system. However, DsRed.T4-NLS seems less than ideal as a reporter to use in conjunction with EGFP-NLS.

We were concerned that the inserted spacer sequences might possess unwanted enhancer activities that complicate the observed reporter expressions. Thus, we sought to see whether the 8 kb spacer (which includes the sequences that makeup the 1, 2, and 4 kb spacers) could drive reporter expression in the pupal abdomen when the pRLGL8 dual transgenes did not include an enhancer. This enhancer-less configuration did not drive any noteworthy expression in the pupal abdomen (Figure S2). While this observation does not rule out the possibility of the spacer possessing enhancer activities in other cell types or developmental stages, it is encouraging that this spacer may generally be lacking regulatory capability and be of broad use to the community studying gene expression regulation in *D. melanogaster*.

A second concern was that placement of the DsRed.T4-NLS reporter gene’s promoter at a distance of 8 kb from the enhancer, created a situation where the enhancer could only activate one promoter at a time, and that there was an enhancer preference for the proximal promoter of the EGFP-NLS reporter gene. To test whether this concern is a real problem, we deleted the proximal *Hsp70* promoter. The absence of this promoter resulted in an inability of the dimorphic element to activate EGFP-NLS expression, while no noteworthy expression activation was observed for DsRed.T4-NLS from the remaining distal promoter (Figure S2). These results suggest that promoter competition is not having unwanted effects on the utility of this vector system. Collectively, our results support the utility of this system for studying proximal and distal gene expression regulation simultaneously.

### The differing abilities of enhancers to regulate a distal reporter gene

**Figure 4:**
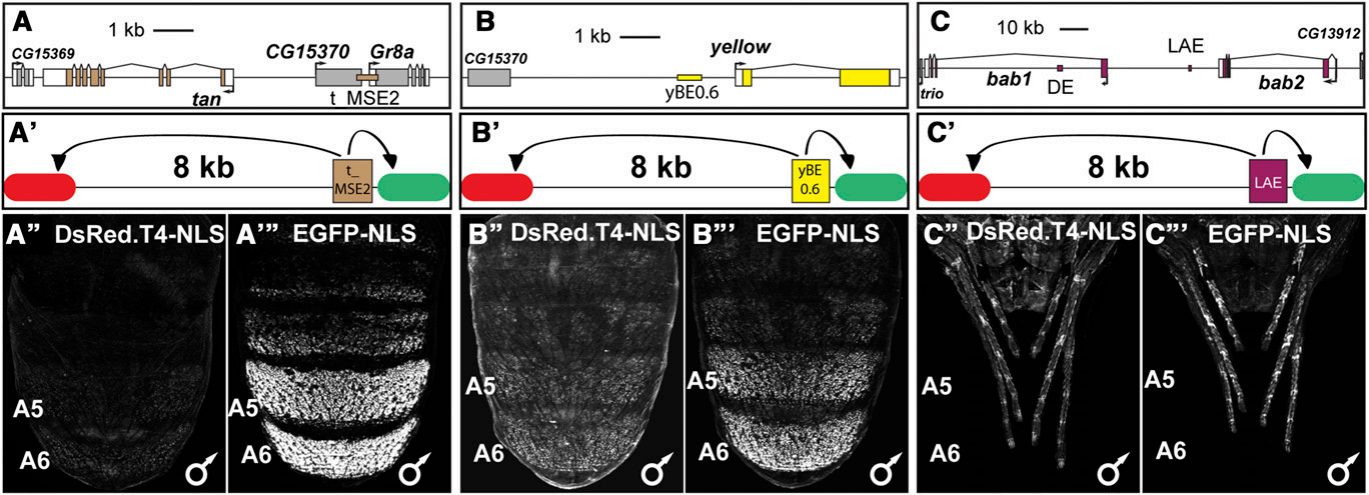
Test of long distance regulatory activity for several *D. melanogaster* enhancers. (A) *tan* gene locus, t_MSE2 is situated between *CG15370* and *Gr8a* genes and 3,239 base pairs (bp) from the *tan* gene promoter. (B) In the *yellow* gene locus, the yBE0.6 located 811 bp proximal of *yellow* promoter. (C) Within the *bab* gene locus is the leg and antennal enhancer (LAE). The LAE is located 27,907 bp and 46,850 bp from paralogous *bab1* and *bab2* gene promoters respectively. (B’-F’) Schematics of the evaluated reporter transgenes. Here, the only difference between the reporter transgenes being compared is which enhancer was included between the distal (8 kb spacer separating from the enhancers) DsRed.T4.NLS (red oval) reporter and the enhancer-proximal EGFP-NLS (green oval) reporter. (A’’ and A’’’) The regulatory function of the t_MSE2 drives little to no expression from the distal reporter and robust expression from the proximal reporter. (B’’ and B’’’) The regulatory function of yBE0.6 drives low levels of distal reporter and robust expression of the proximal reporter. (C’’ and C’’’) The LAE drives similar levels of expression for the proximal and distal reporters.

While the dimorphic element lacked the ability to impart its regulatory activity on an *Hsp70* promoter at an 8 kb distance, it remained a possibility that other enhancers possessed differing long-range activating abilities. Thus, we tested three additional *D. melanogaster* enhancers that are active during pupal development (Figure 4). We first tested the tan_Male Specific Element 2 (t_MSE2), which drives reporter gene expression in the A5 and A6 dorsal abdomen segments of male pupae (Camino *et al.* 2015). The t_MSE2 resides ∼3 kb from the promoter of the *tan* gene (Figure 4A), where it is situated between the *Gr8a* and *CG15370* genes that it is not known to regulate. This genomic arrangement suggests that a mechanism exists by which the t_MSE2 specifically interacts with the *tan* gene. When the t_MSE2 was included in the pRLGL8 transgene, we found that it drives proximal reporter expression in the male A5 and A6 segments (Figure 4A’’’). Similar to the dimorphic element, the t_MSE2 had little to no ability to activate expression of the distal reporter (Figure 4A’’). At least two explanations exist for this outcome. One being that a remote control element exists in a sequence outside of the t_MSE2 enhancer. The second is that a tethering element located proximal to the *tan* gene promoter exists which is needed for the t_MSE2 to activate expression over a distance.

Next, we tested a minimal Body Element (yBE0.6) enhancer of the gene *yellow*, which drives expression of an adjacent reporter transgene in the posterior dorsal abdominal segments of the male abdomen during *D. melanogaster* pupal development. This pattern mimics the endogenous expression of the *yellow* gene at this time point (Camino *et al.* 2015). The yBE0.6 sequence resides 811 base pairs (bp) upstream of the *yellow* gene’s promoter (Figure 4B). In the pRLGL8 construct, the yBE0.6 drove the proximal EGFP-NLS reporter in the male abdomen (Figure 4B’’’). This enhancer also activated the distal reporter gene, albeit with expression levels noticeably weaker than that occurring from the proximal reporter gene (compare Figure 4B’’ to 4B’’’). This outcome suggests that within this enhancer’s sequence of 632 bp resides a motif or motifs that can impose some regulatory activity upon a promoter that is displaced by 8 kb. The existence of such a motif might be identifiable by subjecting the yBE0.6 to scanning mutations and dissecting any motifs as sequences that result in a distal attenuation outcome when mutated (Figure 2C). It is possible that this distal activity results from this enhancer’s interacting transcription factors being better suited for long-range activation in this transgenic context than those for the other tested enhancers.

Finally, we tested the Leg and Antennal Enhancer (LAE), which is an element that resides in the intergenic region between the paralogous *bab1* and *bab2* genes (Baanannou *et al.* 2013) and drives expression of these paralog genes in the leg and antenna of *D. melanogaster*. This enhancer resides ∼30 kb and ∼50 kb from the *bab1* and *bab2* gene promoters respectively (Figure 4C). The LAE can drive the expression of the proximal EGFP-NLS reporter in the developing legs of transgenic pupae (Figure 4C’’’). Interestingly, this enhancer also drove expression of the distal DsRed.T4-NLS reporter in a similar pattern and levels (Figure 4C’’). This indicates that this enhancer encodes a regulatory activity that can be conveyed to a heterologous promoter over an 8 kb distance. Of the enhancers we tested, the LAE provides the best candidate for the identification of an RCE motif or motifs.

### Test of flanking enhancer sequences and promoter type on long distance gene regulation

**Figure 5:**
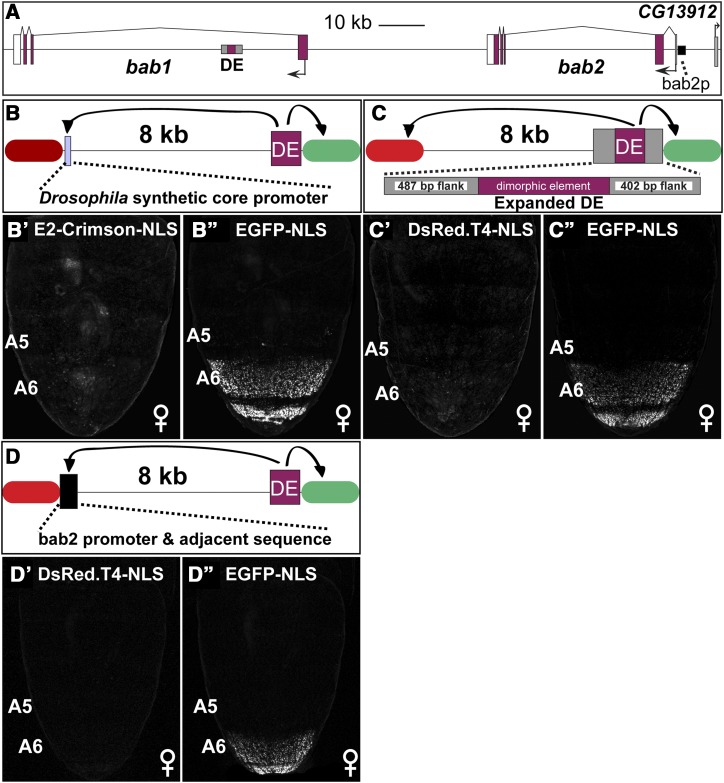
An optimized promoter and additional flanking sequences are insufficient to confer long-distance enhancer-promoter communication. (A) *bab* locus showing the relative position of the dimorphic element (DE), its surrounding flank sequences (gray rectangles), and *bab2* promoter region and adjacent sequence (black rectangle). (B-D) Schematics of the evaluated reporter transgenes, with the distal reporter genes represented as red ovals, and the proximal reporter genes as green ovals. (B) The distal *Hsp70* promoter was replaced by the *Drosophila* synthetic core promoter. (C) The dimorphic element core was expanded to possess additional flanking endogenous *bab* locus sequence. (D) The distal *Hsp70* promoter was replaced by the Drosophila melanogaster bab2 promoter region and adjacent sequence. (B’ and B’’) The dimorphic element (DE) was unable to activate the expression of a (B) distal reporter that possessed the *Drosophila* synthetic core promoter. (C) The additional 487 bp and 402 bp of endogenous sequence to the DE (C’ and C’’) did not improve its ability to drive expression of the distal reporter gene. (D) The replacement of the distal *Hsp70* promoter with the presumptive *bab2* promoter and 5′ sequence (D’ and D’’) did not improve the DE’s ability to drive distal reporter gene expression.

One possible reason why the dimorphic element failed to mediate long-range activation of the distal reporter gene was that the *Hsp70* promoter lacked an element or elements necessary for interacting with it. Thus, we replaced the distal *Hsp70* promoter with the *Drosophila* synthetic core promoter (DSCP) (Pfeiffer *et al.* 2008). The DSCP was created as a minimal promoter that would be capable of interacting with enhancers from diverse *D. melanogaster* genes and drive reporter transgene expression. The DSCP contains a TATA box, initiator element, downstream promoter element and a motif ten element (Pfeiffer *et al.* 2008). This promoter’s initial use was as part of traditional transgenes in which enhancers are situated adjacent to this promoter, thus precluding any need for long distance communication. In our dual reporter system (Figure 5B), the dimorphic element failed to activate expression of the DSCP E2Crimson-NLS transgene when the distance between the enhancer and promoter was 8 kb (compare Figure 5B’ to 5B’’). The failure of the dimorphic element to activate expression of a distal reporter transgene with the *Hsp70* or DSCP promoter might be explained by the dimorphic element having been truncated to exclude sequences encoding a remote control element. To test this hypothesis, we added ∼450 bp of the endogenous *bab* locus sequence that flanks each side of the minimal dimorphic element (called expanded DE, Figure 5C). However, this expanded enhancer version failed to convey the regulatory activity of the dimorphic element to the distal *Hsp70* promoter (compare Figure 5C’ to 5C’’). This indicates that other *cis*-acting sequences are needed for the dimorphic element to activate the expression of a gene positioned at a distance.

It seemed reasonable to suspect that the dimorphic element might need to interact with a promoter or promoter-proximal sequence that is present in the endogenous *bab1* and *bab2* loci to drive reporter expression in the female pupal abdomen. To test this possibility, we replaced the distal *Hsp70* promoter with a 1,157 bp sequence that includes the presumptive *bab2* promoter and adjacent 5′ sequence (bab2p, Figure 5D). While typical expression output occurred for the proximal promoter, no noteworthy expression was observed from the endogenous distal promoter region (Compare Figure 5D’ to 5D’’). This result indicates that we have not yet identified the minimal set of sequences sufficient for the long-distance regulatory activity of the dimorphic element enhancer.

### Identifying a fluorescent reporter to use in conjunction With EGFP-NLS

**Figure 6:**
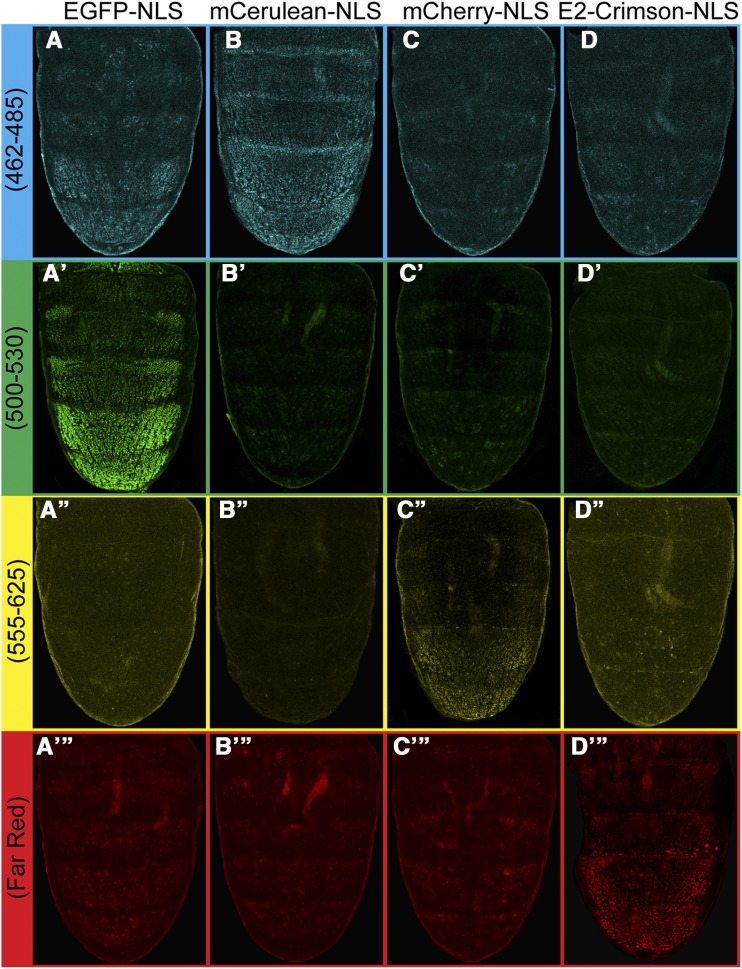
Comparison of fluorescence properties of various fluorescent reporters when regulated by an enhancer. Transgenic *D. melanogaster* were made that possessed the yBE0.6 enhancer driving the expression of fluorescent reporters with an *Hsp70* minimal promoter. The reporters included (A-A’’’) EGFP-NLS, (B-B’’’) mCerulean-NLS, (C-C’’’) mCherry-NLS, and (D-D’’’) E2-Crimson-NLS. For all transgenic fluorescent reporters, male pupae were imaged at settings optimized for blue, green, red, and far-red light.

While the red fluorescence of DsRed.T4-NLS worked well as a readout of long-distance transcriptional activation, it was less than ideal for monitoring of the proximal EGFP-NLS reporter’s expression. Thus, we sought to identify a more suitable fluorescent reporter protein to pair with EGFP-NLS. We synthesized the coding sequences for several fluorescent proteins in-frame with the coding sequences for a C-terminal Tra nuclear localization signal (Hedley *et al.* 1995). Our goal was to identify a nuclear-localized fluorescent protein with easily detectable signal that does not noticeably overlap with the signal from EGFP-NLS. The fluorescent proteins we selected and tested were mCherry-NLS, mCerulean-NLS, and E2-Crimson-NLS (Figure 6). We suspected that fluorescence of mCherry-NLS would be best-detected using modestly red-shifted settings and to a lesser extent far-red settings, whereas the mCerulean-NLS and E2-Crimson-NLS would only be detected using blue shifted and far-red shifted settings, respectively. E2-Crimson-NLS was of high interest as the published emission spectrum for E2-Crimson is the most distinct from that for EGFP (Strack *et al.* 2009). However, we did not know whether this protein results in an immature green light emitting form as seen for DsRed (Baird *et al.* 2000) and DsRed.T4-NLS (Barolo *et al.* 2004) (Figure 3).

To test the fluorescent properties of the newly synthesized reporters, we coupled them to an *Hsp70* minimal promoter and the yBE0.6 enhancer that drives a male-limited pattern of expression in the pupal dorsal epidermis of the A5 and A6 abdomen segments of transgenic *D. melanogaster*. Optimal excitation and emission settings were identified for each of the four fluorescent reporters (Table 2), and transgenic pupae with each of the single fluorescent reporters were imaged at the optimal settings for all reporters tested (Figure 6). While little to no fluorescence was detected from EGFP-NLS when using the red (mCherry) and far-red (E2 Crimson) settings, the male A5 and A6 expression was seen with the blue-shifted settings for mCerulean (Figure 6A-6A’’’). This outcome indicates that EGFP-NLS and mCerulean-NLS are not an ideal pair of fluorescent proteins to utilize in our dual reporter transgene experiments, even though the mCerulean-NLS signal was only observed with the blue shifted settings (Figure 6B-6B’’’).

mCherry is a commonly utilized fluorescent protein in biological experimentation, and it possesses a red-shifted emission spectra compared to EGFP. E2-Crimson has a far-red emission spectra, though it has only recently been developed and characterized (Strack *et al.* 2009) and to our knowledge it has not been used previously in fruit flies. We found that our mCherry-NLS and E2-Crimson-NLS reporters had noteworthy expression when using the red-shifted and far red-shifted settings respectively (Figure 6C-6C’’’ and 6D-6D’’’). While both reporter proteins seemed compatible for use with EGFP-NLS in dual reporter experiments, we opted to further utilize E2-Crimson-NLS as its signal seemed easier to detect among replicate specimens. These results also demonstrate the potential that EGFP-NLS, mCherry-NLS, and E2-Crimson-NLS have in future applications that require three reporters.

### EGFP-NLS and E2-Crimson-NLS provide specific read outs on proximal and distal reporter gene expression

**Figure 7:**
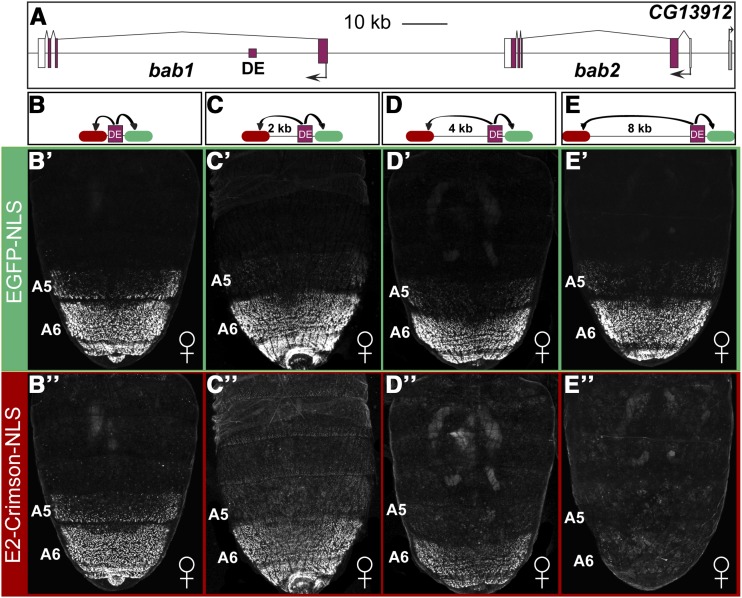
E2-Crimson-NLS and EGFP-NLS reporters provide optimal readouts of distal and proximal regulatory activities of an enhancer. (A) *bab* locus showing the relative position of the dimorphic element (DE) core enhancer from the promoters (black arrows) for the *bab1* and *bab2* genes. (B-E) Schematics of the evaluated reporter transgenes. Here, the distance of the dimorphic element from the E2-Crimson-NLS (red oval) reporter was altered by the inclusion of spacer sequence, while the EGFP-NLS (green oval) reporter’s position did not change. The regulatory activities of the dimorphic element (purple square) on proximal and distal promoters were evident when using EGFP-NLS and E2-Crimson-NLS reporters. (B’ and B’’) At equal spacing from the reporter genes, the dimorphic element drives identical patterns and comparative levels of EGFP-NLS and E2-Crimson-NLS reporter expression. (C-E) When spacer sequence of 2, 4, and 8 kb were situated between the enhancer and the E2-Crismon-NLS reporter gene, (C’-E’) the expression observed from the more proximal EGFP-NLS was consistent. (C’’-E’’) Conversely, expression seen from the E2-Crimson-NLS reporter declined proportional to the length of spacer sequence.

With E2-Crimson-NLS having far-red (FR) fluorescent excitation and emission spectra distinct from EGFP-NLS, we sought to see whether it performs equally well in a dual reporter transgene context. Thus, we replaced the DsRed.T4-NLS coding sequence in the pRLGL0, 2, 4, and 8 kb vectors that possess the dimorphic element enhancer with the E2-Crimson-NLS coding sequence (Figure 7B-7D). When these dual reporters (pFRGL0, 2, 4, and 8+DEcore) were site-specifically integrated in *D. melanogaster*, we observed a progressive decrease in far-red fluorescence as the E2-Crimson-NLS reporter was moved further distal to the dimorphic element (Figure 7B’’-7E’’). However, the green fluorescence remained more consistent (Figure 7B’-7E’), suggesting that in this dual reporter system, green light is predominately due to the EGFP-NLS reporter and far red light from the E2-Crimson-NLS reporter.

## Discussion

We have developed an optimized dual reporter transgene system in *Drosophila* that permits the simultaneous comparison of an enhancer’s capability to activate a distal or proximal promoter sequence region. Using a well-studied enhancer involved in abdominal pigmentation, we found that this sequence can similarly activate two fluorescent reporter transgenes when they are at equal proximal positions. However, as one of the reporters is placed progressively further away from the dimorphic element (starting at 1 kb), the level of expression declines until it can no longer be observed (at a distance of 8 kb). Tests of three additional *D. melanogaster* enhancers revealed a range of capabilities to activate a distal promoter over long distances. Thus, different enhancers possess distinct capabilities to activate gene expression from a distally located heterologous promoter. For one tested enhancer its inability to activate the distal reporter gene at an 8 kb distance indicates that the enhancer’s *in vivo* function must be mechanistically complex, requiring sequences beyond its proximal promoter or enhancer-adjacent sequences. Using a combination of fluorescent proteins that we optimized for maximal spectral separation, this system will promote an understanding of the phenomenon of long-distance communication between enhancers and promoters.

### When does gene regulation become long distance?

An initial question we sought to pursue was the effect of distance between an enhancer and a distal reporter transgene on its regulative activity. To answer this question, we chose the dimorphic element of the *D. melanogaster bab* locus as our test case. The endogenous function of this enhancer is to control the female-specific expression of the *bab1* and *bab2* genes in the A5-A7 segments of the pupal abdomen (Williams *et al.* 2008; Rogers *et al.* 2013). This CRE is situated in the large first intron of the *bab1* gene, at a distance of ∼16 and ∼92 kb from the promoters for the *bab1* and *bab2* genes, respectively. Since this enhancer is naturally positioned at a great distance from its target promoters, we suspected that it may possess a “remote control element” (Swanson *et al.* 2010) that enables it to impart its regulatory activity over a great distance. To our surprise, we found that this enhancer’s ability to activate the expression of a heterologous promoter began to decline even when the distance of separation was 1 kb (Figure 3). At a distance of 4 kb, its activity was further reduced, and at 8 kb we saw little to no expression from the distal reporter gene. Thus for the dimorphic element, and in this transgenic context, 8 kb was enough distance to sufficiently impede reporter gene expression activation. This 8 kb distance was also sufficient to impede the *D. melanogaster* t_MSE2 enhancer from imparting its male-specific regulatory activity (Camino *et al.* 2015) on a heterologous promoter (Figure 4). The endogenous position of this enhancer is between two genes that it is not known to regulate, and at a distance of ∼3 kb from the *tan* gene’s promoter. We also tested the activity of the yBE0.6 and LAE enhancers for the ability to activate the distal reporter at an 8 kb distance (Figure 4). The endogenous position of the yBE0.6 is ∼1 kb upstream of the *yellow* gene promoter from which it drives a male-specific pattern of pupal abdomen expression (Camino *et al.* 2015). The LAE is located ∼28 kb from the *bab1* promoter and ∼47 kb from the *bab2* promoter, from which the enhancer drives leg and antennal expression of the two paralogous *bab* genes (Baanannou *et al.* 2013). Interestingly, the yBE0.6 was able to drive a low-level of expression from the distal reporter even though this enhancer is naturally located at a close distance to its promoter. In contrast to the dimorphic element, the LAE was able to robustly activate the expression of a distal reporter.

Our results have several noteworthy implications. First, it is clear that enhancers can possess differing abilities to activate gene expression from a minimal promoter when at a distance of 8 kb. While many are at an even greater distance *in vivo* (Kvon *et al.* 2014), this transgene context with a displacement of up to 8 kb appears to provide a useful compromise for mechanistic studies. Second, 3 of 4 enhancers tested indicated that 8 kb is an effectively long-distance for a reporter transgene. In a seminal study, it was shown that the sparkling enhancer possessed a “remote control element” sequence that was necessary to impart the cone-cell pattern of gene expression regulation on a reporter transgene at a distance of ∼0.8 kb (Swanson *et al.* 2010). For the dimorphic element, we observed only a subtle decrease in expression at a distance of 1 kb. Thus, greater distances must be tested to identify sequences sufficient to confer long- distance activation. However, care must be taken in selecting spacer distance, as we found cloning to be more difficult into the vector containing the 8 kb spacer (∼20 kb total plasmid size).

### Differing abilities of enhancers to interact with a distal heterologous promoter

The *Hsp70* promoter is commonly utilized in reporter transgene experiments where an enhancer is situated immediately adjacent to it (Barolo *et al.* 2004; Rebeiz and Williams 2011; Rogers and Williams 2011). In this study, we found that this minimal promoter can be effectively regulated over a distance by some but not all enhancers. One possible explanation for these outcomes is that some enhancers, like the LAE (Figure 4), possess a remote control element, whereas others, like the dimorphic element, do not. For the dimorphic element, we suspected that when it was first characterized in traditional reporter transgene studies, that long-distance regulation was not required and perhaps the remote control element was removed during the process of identifying the minimal sufficient sequence needed to activate a proximal reporter transgene (Williams *et al.* 2008). However, when we restored 487 and 402 base pairs of endogenous flanking sequence to the sides of the minimal dimorphic element, we saw no noteworthy improvement in the ability of this larger sequence to activate the 8 kb displaced distal reporter (Figure 5). This suggests that either a remote control element exists but in more distant *bab* locus sequence, or that the dimorphic element possesses a remote control element which cannot interact with the minimal *Hsp70* promoter. To test this latter possibility, we separately replaced the distal *Hsp70* promoter with the Drosophila Synthetic Core promoter, called the DSCP (Pfeiffer *et al.* 2008), and a 1 kb sequence that includes the presumptive *bab2* promoter and adjacent sequence. However, we found that the dimorphic element could not activate expression from either of these promoters at an 8 kb distance. These results suggest that long distance regulation by the dimorphic element requires *cis*-acting sequences that we have yet to identify. These may include “tethering element” (Calhoun and Levine 2003) which may lie at distinct locations within the *bab* locus.

Many searches for enhancers often begin by testing large pieces of genomic DNA (≥3kb) for the ability to activate expression of a heterologous promoter in a reporter transgene assay. Our results suggest that this methodology is at risk for failing to identify regulatory activities when these sequences are at a distance to an ill-suited promoter. This justifies the additional examination of weak activities detected in larger genomic fragments, as the tested region may lack elements for long-range interactions. The existence of these features raises the conundrum that heavily dispersed elements that mediate long-range interactions may exist and be exceedingly difficult to find. Integrating these into a system such as Red Light/Green Light may require unbiased high throughput/genomic approaches such as Hi-C, 3C, and 5C (Dostie *et al.* 2006; Dekker and Misteli 2015). The current challenge of such approaches is that they tend to require large numbers of cells, whereas developmental enhancers are usually active in only small portions of a tissue of interest. Studies that use dual reporter systems to validate the *in vivo* significance of topologically associated domains will begin to provide meaning and biological context to these data.

### Mapping cis-acting sequences required for long distance gene regulation

Our motivation for developing Red Light/Green Light was to provide a means to identify the DNA sequences involved in mediating gene regulation between a distantly located enhancer and its target promoter. What has been previously referred to as remote control (Swanson *et al.* 2010) and tethering (Calhoun *et al.* 2002) elements. For the t_MSE2 and dimorphic element, we must first identify the promoter and *cis*-acting sequences necessary for long-distance regulation. However, the LAE provides an opportunity to seek and characterize a remote control element. Future studies should subject the LAE to mutations to identify the RCE element as the sequence that when mutated results in attenuated distal reporter expression (Figure 2). Discovery of such an element would allow for the subsequent identification of the proteins that directly interact with the remote control element. Success here should serve as a needed entry point to understand how enhancers encode information that facilitates long-distance gene expression activation.

### Evolutionary implications of long-range enhancer-promoter interactions

A major theme in the evolution of development is that changes in gene expression, driven by non-coding mutations play significant roles in generating morphological traits (Wray 2007; Carroll 2008). We suggest that changes to these long-range interactions may be quite significant to the evolution of gene expression more generally. First, increases and decreases in expression are frequently associated with morphological traits (Stern and Orgogozo 2008; Martin and Orgogozo 2013). It may be that these changes are mediated by adjusting the strength of long-range interactions rather than simply strengthening or weakening binding sites for activators and repressors. Such an alteration to enhancer-promoter communication would not be detected in traditional reporter systems. Second, one major mechanism for the origin of enhancer sequences is through changes in enhancer promoter specificity: a preexisting enhancer may evolve novel interactions with a different promoter to confer a new expression pattern upon the target gene (Rebeiz *et al.* 2011; Rebeiz and Tsiantis 2017). Finally, a major posited source of novelty is the evolution of new enhancers, which raises the question of how their long-range interactions first originate. In tightly packed genomes, it may be that remote control elements are relatively pleiotropic, interacting with multiple enhancers. Indeed, their degree of pleiotropy will likely shape how often they participate in evolutionary modifications. Reporter assays represent a crucial line of evidence used to resolve the functional implications of gene regulatory mutations (Rebeiz and Williams 2011). The Red Light/Green Light system will provide a much-needed tool to probe the extent to which non-coding mutations alter long-range interactions during evolution.
